# Genetic Diversity of Fresh Maize Germplasm Revealed by Morphological Traits and SSR Markers

**DOI:** 10.3390/genes16101138

**Published:** 2025-09-25

**Authors:** Suying Guo, Xin Zheng, Shuaiyi Wang, Yuran Ai, Rengui Zhao, Jinhao Lan

**Affiliations:** 1 College of Agronomy, Qingdao Agricultural University, Qingdao 266109, China; ggygsy@163.com (S.G.); xinz1106@163.com (X.Z.); 2 College of Agronomy, Jilin Agricultural University, Changchun 130118, China; 3188055501@mails.jlau.edu.cn (S.W.); 20230762@mails.jlau.edu.cn (Y.A.); renguiz@jlau.edu.cn (R.Z.)

**Keywords:** fresh maize, phenotype, SSR markers, genetic distance, genetic diversity

## Abstract

Background: This study aims to systematically evaluate the genetic divergence among 200 fresh maize inbred lines using both phenotypic and molecular markers, and to compare the efficacy of these two approaches for genetic classification. Methods: Phenotypic clustering analysis was conducted based on eight key agronomic traits, including plant height and ear length. Additionally, molecular analysis was performed using 40 Simple Sequence Repeat (SSR) primer pairs, resulting in the generation of 230 polymorphic alleles. The polymorphism information content (PIC) was calculated to evaluate the discriminatory power of the markers. Results: Phenotypic analysis categorized the inbred lines into four groups, comprising 25, 38, 97, and 40 lines, respectively, with benchmark lines distributed across Groups I and III. SSR analysis revealed a high level of genetic diversity, with an average of 5.75 alleles per locus and a mean polymorphic information content (PIC) of 0.70. Molecular grouping further divided the population into four distinct clusters, representing 26.5%, 51.0%, 14.0%, and 8.5% of the total, which exhibited different distribution patterns compared to the phenotypic grouping. The distribution of benchmark lines across various molecular groups confirmed their genetic divergence. Conclusions: SSR-based clustering demonstrated superior robustness and reliability compared to phenotypic grouping for genetic discrimination. These findings confirm the substantial genetic diversity present in fresh maize inbred lines and support the preferential use of SSR markers in maize breeding programs for precise genetic characterization.

## 1. Introduction

Fresh maize is a specialty type that is highly valued for its unique flavor and nutritional richness. Both its global cultivation area and market demand have experienced remarkable growth. Agricultural restructuring plays a significant role in increasing farmers’ income and meeting consumers’ demands for diverse agricultural products. Fresh maize is primarily categorized into three types: sweet corn, waxy corn, and sweet-waxy corn. From a genetic classification perspective, sweet corn can be further divided into normal sweet maize, super sweet maize (which includes *sh2*, *bt1*, and *bt2* genotypes), and enhanced sweet maize (*se*) [1]. Waxy corn, also known as glutinous corn or waxy maize, is characterized by an endosperm starch composed almost entirely of amylopectin. This unique composition imparts a distinctive sticky texture and a soft, chewy consistency when cooked. In comparison to sweet corn, waxy corn exhibits a lower sugar content, typically ranging from 5% to 10%. This type originated from a recessive mutation in the *Wx* gene located on the short arm of chromosome 9 in common maize (*Zea mays* L.), which results in its distinct glutinous properties upon cooking. Based on kernel coloration, waxy corn can be classified into several types: white waxy corn, yellow waxy corn, purple waxy corn, black waxy corn, and multicolored waxy corn [2]. Among these, white and yellow waxy corn types are the most commonly consumed in daily life. Sweet-waxy corn is a hybrid type that combines the desirable traits of both sweet corn and waxy corn. This innovative type produces ears that contain both sweet and glutinous kernels on the same cob, thereby satisfying consumer demand for a dual-texture experience that offers both sweetness and chewiness [3,4].

In 2022, the cultivation area of fresh maize in China expanded significantly, reaching over 1.666 million hectares. This growth trajectory has persisted, further solidifying China’s position as the global leader in maize production. This consistent expansion is driven by steadily increasing yields and the maturation of cultivation technologies. Consequently, China has successfully established itself as the world’s largest producer of fresh maize, thereby securing a prominent position in the global fresh maize market [5].

The development of fresh maize types is essential for agricultural advancement. In hybrid breeding programs, the selection of appropriate parental lines is critical. Breeders generally cross elite parental lines that exhibit desirable traits, including early maturity, high yield, and superior quality, to create high-performance hybrid types.

Phenotypic trait evaluation, commonly referred to as morphological markers, serves as a method for assessing plant genetic diversity through the observation and statistical analysis of external morphological characteristics [6]. The investigation and classification of phenotypic traits represent the most intuitive approach. This method has been extensively utilized in preliminary species assessments [7,8,9]. To date, phenotypic studies have been conducted on various crops, including rice [10], wheat [11], maize [12], and mung bean [13,14]. However, phenotypic markers demonstrate significant limitations: the number of measurable traits is restricted, their expression is influenced by environmental factors, leading to genetically unstable manifestations, and they may obscure inherent genetic variation among alleles, as similar phenotypic expressions can arise from different alleles. With the advancements in molecular marker technologies, DNA-based detection methods have increasingly been adopted as standard practices by official institutions, such as the International Union for the Protection of New Varieties of Plants (UPOV). This trend is attributed to their higher detection efficiency, greater polymorphism, and enhanced genetic stability [15].

The relatively limited genetic diversity in Chinese maize germplasm primarily results from a long-term overreliance on four key inbred lines: The repeated utilization of Reid, Lancaster, Tangsipingtou, and Lüda Red Cob in breeding programs has resulted in a narrowed genetic base [16]. This genetic bottleneck not only constrains the discovery and exploitation of favorable alleles, thereby impeding the advancement of maize breeding in China, but it also contributes to cultivar degeneration and reduced stress tolerance. Ultimately, this undermines the resilience of the agricultural system to natural disasters [17]. Consequently, the enrichment and innovation of germplasm through the accumulation of novel genetic resources has emerged as a critical objective for maize breeding research and development in China.

This study systematically evaluates the genetic diversity of fresh maize germplasm resources in China and investigates the difference between morphological markers and SSR molecular marker analyses. This research hypothesizes that, despite a potentially narrow overall genetic base in existing germplasm, strong selection for specific consumer-oriented traits, such as sweetness and waxiness, has driven divergence at key genomic loci. Consequently, we propose that the three flavor types-sweet corn, waxy corn, and sweet-waxy corn-may represent distinct genetic groups, defined more by these selected regions than by overall genetic variability. By integrating both phenotypic and molecular-level data analyses, we aim to establish a comprehensive phenotype-genotype database comprising 200 inbred lines. This initiative will elucidate the characteristics of population structure and genetic relationships, ultimately facilitating the identification of parental materials with breeding potential. Furthermore, it will provide a theoretical foundation for the selection of hybrid combinations in fresh maize.

## 2. Materials and Methods

### 2.1. Materials

A total of 200 fresh maize inbred lines, representing a diverse range of genetic backgrounds, were collected from various geographical regions and uniformly designated as GM001 to GM200. This collection includes four benchmark inbred lines: GM002 (Ye478), GM003 (Dan340), GM007 (Mo17), and GM171 (Qi319), which serve as standard testers. The remaining 196 accessions represent newly acquired germplasm materials sourced from various production areas. The experimental materials utilized in this study were procured from the following suppliers: primers from Sangon Biotech (Shanghai) Co., Ltd., Shanghai, China, and other reagents from Yicheng Chemical Reagent Business Department, Tai’an, China.

### 2.2. Methods

#### 2.2.1. Field Experiment Design, Phenotypic Trait Investigation, and Seed Character Evaluation Methods

In mid-June 2022, all 200 experimental materials were planted at two locations: the Mizhou Seed Industry Experimental Base in Zhucheng, Weifang, Shandong Province (36°23′ N, 119°24′ E) and the experimental field in Pingdu, Qingdao, Shandong Province (36°47′ N, 119°58′ E). The trial employed a non-replicated design, with each plot consisting of two rows containing twenty plants per row. The planting configuration maintained a spacing of 60 cm between rows and 30 cm between plants within each row. Throughout the entire growth cycle of the fresh maize inbred lines, field management practices—including intertillage, irrigation and fertilization regulation, as well as pest and disease control—were strictly implemented in accordance with local commercial production standards. The total experimental area at each site was approximately 800 square meters. From the central area of each accession’s plot, five representative plants exhibiting uniform growth and free from pests or diseases were systematically selected.

During the experimental period, three categories of phenotypic traits were systematically investigated: field performance traits, agronomic traits, and yield component traits. Field performance traits encompass the observable and measurable characteristics of crops during growth in field conditions. These traits are crucial for evaluating plant growth status, yield potential, stress resistance, and quality parameters. These traits serve as fundamental indicators for monitoring crop development and adaptation. Agronomic traits represent measurable characteristics associated with crop growth dynamics, yield formation processes, and quality attributes throughout the production cycle. As critical benchmarks for assessing cultivar performance and the effectiveness of cultivation management, these traits provide essential guidance for agricultural production practices aimed at enhancing both the quantity and quality of yield. Yield-component traits encompass all characteristics that directly or indirectly influence crop productivity. These critical traits predominantly determine the final yield output and thus serve as primary objectives in crop genetic improvement and varietal development programs. The measurement and optimization of these traits are essential for achieving breeding targets and enhancing agricultural productivity. In this study, plant height, ear height, tassel length, and the number of tassel branches are classified as field performance traits. Ear length and ear diameter are categorized as agronomic traits. The number of kernel rows and the number of kernels per row are identified as yield-component traits.

#### 2.2.2. Statistical Methods for Phenotypic Traits

Two hundred fresh maize inbred lines were classified into distinct groups through cluster analysis based on eight key phenotypic traits: plant height, ear height, tassel length, tassel branch number, ear length, ear diameter, kernel row number, and kernel number per row. The analysis was conducted using the Unweighted Pair Group Method with Arithmetic Mean (UPGMA) algorithm, implemented in R software (version 4.2.1). This hierarchical clustering approach facilitated the systematic grouping of inbred lines based on their phenotypic similarities. The resulting dendrogram illustrates the genetic relationships among various accessions.

#### 2.2.3. SSR Analysis and Data Processing Methods

(1) In the analysis of genetic diversity, bands obtained through SSR silver staining were scored using a co-dominant scoring system. Homozygous loci were recorded with their corresponding allele fragment sizes (e.g., “215/215”), while heterozygous loci were recorded with both allele fragment sizes (e.g., “215/221”). Missing data were denoted as “9”. This scoring facilitated the construction of a comprehensive database. The genetic similarity coefficient (GS) and genetic distance (GD) between fresh maize inbred lines were calculated using Nei’s formula [18]. The specific formulas are expressed as follows: GS_ij_ = 2N_ij_/(N_i_ + N_j_) and GD_ij_ = 1 − GS_ij_. Here, N_ij_ represents the number of bands shared between inbred lines *i* and *j*, while N_i_ and N_j_ denote the total number of bands present in inbred lines *i* and *j*, respectively. These equations are critical for understanding the genetic similarities and differences among various inbred lines. After completing the calculations, a cluster analysis was performed using the UPGMA method to construct a dendrogram. The polymorphism information content (PIC) of each polymorphic locus was calculated according to the formula proposed by Smith et al. [19]: PIC = 1 − ∑f_i_^2^, where fi represents the allele frequency at locus i.
(2) The IBM SPSS Statistics software (version 27.0) was employed to compare the phenotypic traits of fresh maize inbred lines for significant differences.

## 3. Results

### 3.1. Phenotypic Cluster Analysis

From June to September 2022, eight phenotypic traits were examined in 200 fresh maize inbred lines: plant height, ear height, tassel length, tassel branch number, ear length, ear diameter, kernel row number, and kernel number per row. Statistical analyses of the agronomic traits presented in Table 1 revealed significant genetic diversity across all measured characteristics. For the phenotypic cluster analysis, the average values of the eight traits from both locations were utilized (refer to Appendix A). The results demonstrated substantial variation, thereby providing a robust foundation for further assessments of genetic diversity and breeding applications.

**Figure 1:**
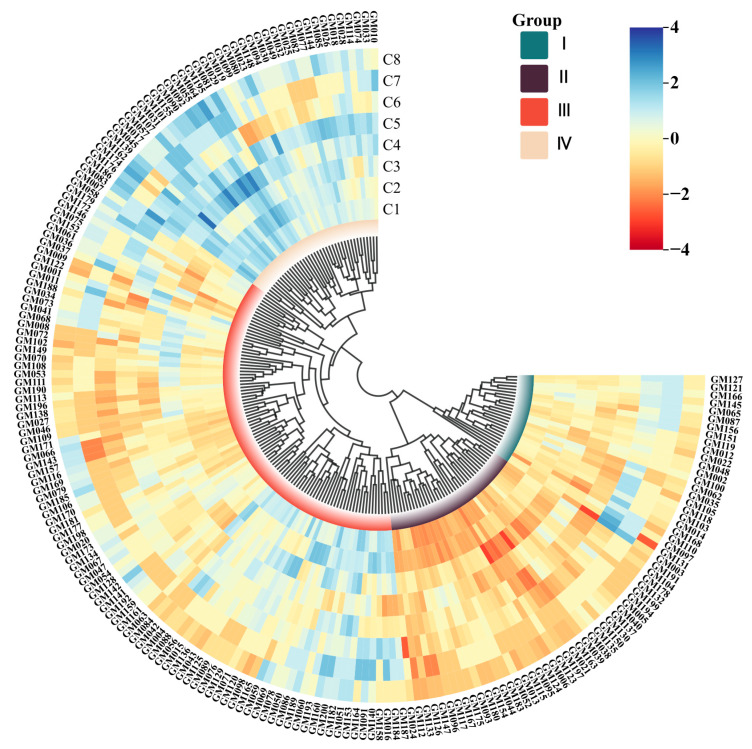
Phenotypic clustering diagram of 200 fresh maize inbred lines. The four major clusters are labeled with Roman numerals (I–IV). The scale bar represents branch lengths in units of genetic distance. Negative values on the scale are mathematical artifacts resulting from the tree-rooting process and have no biological significance. The genetic distance between any two inbred lines is proportional to the sum of the horizontal branch lengths along the path connecting them. C1: Plant height, C2: Ear height, C3: Tassel length, C4: Tassel branch number, C5: Ear length, C6: Ear diameter, C7: Kernel row number, C8: Kernel number per row.

The UPGMA (Unweighted Pair Group Method with Arithmetic Mean) cluster analysis was conducted using the comprehensive data presented in Table A1, with the results illustrated in Figure 1. This analysis demonstrated that the 200 fresh maize inbred lines were categorized into four primary clusters: Cluster I comprised 25 inbred lines, accounting for 12.50% of the total sample; Cluster II included 38 inbred lines, representing 19.00% of the total sample; Cluster III contained 97 inbred lines, constituting the largest proportion at 48.50%; and Cluster IV consisted of 40 inbred lines, making up 20.00% of the total sample. The detailed results of the phenotypic cluster analysis are presented in Table 2. This classification highlights significant phenotypic variation among the fresh maize inbred lines, with Cluster III identified as the most predominant group. The observed distribution patterns indicate potential genetic relationships and phenotypic diversity within the germplasm collection.

The results of the phenotypic cluster analysis indicated that 48.50% of the fresh maize inbred lines were categorized into Cluster III. Notably, the benchmark inbred lines GM002 and GM003 were classified within Cluster I, whereas GM007 and GM171 were assigned to Cluster III. These findings indicate that classification based solely on phenotypic traits has inherent limitations in accuracy, rendering it insufficient for the precise categorization of maize inbred lines. For comprehensive data, please refer to Figure 1.

### 3.2. Genetic Distance Analysis

Genetic distance analysis of 200 fresh maize inbred lines revealed a range of genetic distances from 0.308 to 0.902, with an average genetic distance of 0.657. This average value suggests that there are relatively distant genetic relationships among these inbred lines overall. Through further analysis of the data, the genetic differences among the research materials are clearly delineated in Table 3 and Table 4. As shown in Table 3, the genetic distance between the inbred lines GM013 and GM021 was only 0.308, the smallest among all pairs of inbred lines, indicating that they share closely related genetic backgrounds. Conversely, Table 4 indicates that the inbred lines GM176 and GM184 exhibited the highest genetic distance of 0.902 among all pairs, thereby demonstrating a significant genetic divergence between these two lines.

### 3.3. SSR Genetic Diversity and Cluster Analysis

#### 3.3.1. Polymorphism Analysis of SSR Markers

**Figure 2:**
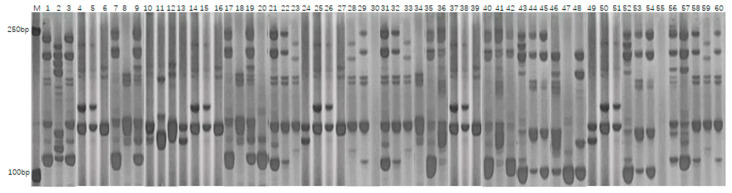
Electrophoregram of PCR amplification products of primer umc2105g1 in 60 fresh maize inbred materials (M: Marker, GM002 to GM061). The marker in Figure 2 is identical to that in Figure 3.

**Figure 3:**
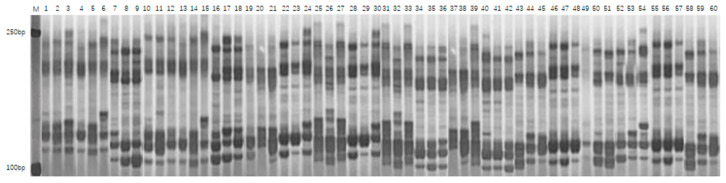
Electrophoregram of PCR amplification products of primer bnlg2305g1 in 60 fresh maize inbred materials (M: Marker, GM122 to GM181). The marker in Figure 3 is identical to that in Figure 2.

PCR amplification was conducted using 40 SSR primer pairs on 200 fresh maize inbred lines, resulting in the detection of a total of 230 polymorphic alleles. The number of alleles identified per primer pair varied from 2 to 9, with an average of 5.75 alleles per locus. The polymorphism information content (PIC) of the primers ranged from 0.30 to 0.83, yielding an average value of 0.70, which indicates a high level of genetic diversity within the population. Notably, the primers umc2105g1 (PIC = 0.82) and bnlg2305g1 (PIC = 0.83) exhibited the highest levels of polymorphism, each identifying nine distinct alleles. The average heterozygosity rate among the inbred lines was 2.1%, indicating a high degree of genetic homozygosity in the materials. Figure 2 presents the electrophoretic separation patterns of PCR products amplified by the primer umc2105g1 across various inbred lines, whereas Figure 3 illustrates those amplified by the primer bnlg2305g1. The observed clear banding polymorphisms and significant differences in fragment sizes reflect the genetic variation among the materials. These results confirm that SSR markers are effective in revealing allelic diversity within the fresh maize population.

The analysis of the SSR marker polymorphism results indicated that the effective number of alleles per locus ranged from 1.71 to 7.92, with an average value of 4.93. This indicates both an uneven distribution of alleles within the population and substantial genetic diversity among the 200 inbred lines. Notably, primer bnlg2305g1 exhibited the highest level of polymorphism (PIC = 0.83), underscoring its remarkable ability to reveal genetic variations among maize materials. Comprehensive data are presented in Table 5.

#### 3.3.2. SSR Marker-Based Cluster Analysis

**Figure 4:**
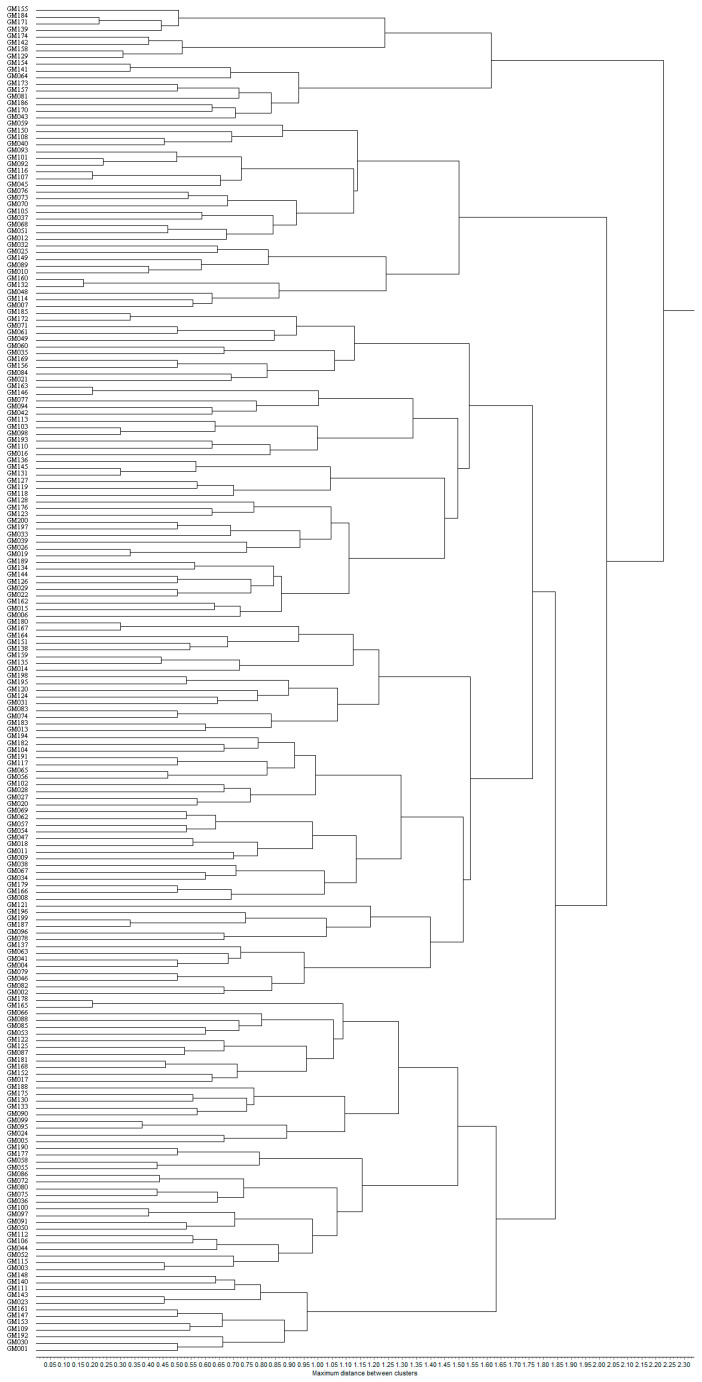
The dendrogram of 200 fresh maize inbred lines with SSR markers.

The Unweighted Pair Group Method with Arithmetic Mean (UPGMA) was utilized to analyze 200 fresh maize inbred lines, resulting in their classification into four distinct clusters. Cluster I consisted of 53 inbred lines, representing 26.50% of the total samples. Cluster II, the largest group, comprised 102 inbred lines, accounting for 51.00% of the total. Cluster III consisted of 28 inbred lines, representing 14.00%, while Cluster IV included 17 inbred lines, making up 8.50% of the total. The detailed clustering results are illustrated in Figure 4, which demonstrates the genetic relationships and population structure among the fresh maize inbred lines.

As illustrated in Table 6, the benchmark selfing lines GM002, GM003, GM007, and GM171 were categorized into distinct taxa. In the study, GM003 (Dan 340) was classified as the first taxon, GM002 (Ye 478) as the second taxon, GM007 (M017) as the third taxon, and GM171 (Qi 319) as the fourth taxon. The four benchmark inbred lines can be accurately classified into their respective lineage taxa. Among all the fresh maize germplasm, GM013 and GM021 exhibited the shortest genetic distance, resulting in their clustering within the second taxon. In contrast, GM176 and GM184 displayed the greatest genetic distance, leading to their categorization into the second and fourth taxa, respectively. Based on the clustering results, it is evident that the clustering analysis utilizing SSR molecular markers can more accurately reflect the genetic associations among various fresh maize germplasm and elucidate their affinities.

## 4. Discussion

The SSR molecular marker technique has been extensively utilized in genetic diversity studies of maize germplasm owing to its high polymorphism, co-dominant inheritance, operational simplicity, and relatively low cost [20,21]. Wang et al. [22] analyzed 35 waxy maize inbred lines and 5 common maize types using SSR markers, successfully classifying them into 4 distinct groups consistent with their pedigree origins. Li et al. [23] characterized 144 sweet maize accessions, identifying seven major genetic clusters and 343 allelic variants, with a range of 4 to 17 alleles per locus and a mean of 8.58 alleles. Huang et al. [24] evaluated 54 sweet maize inbred lines using 56 SSR primers, revealing three primary genetic groups and 155 polymorphic loci. Lu et al. [25] assessed 87 fresh-eating maize inbred lines using 29 SSR markers. Their analysis revealed four genetic clusters and identified a total of 180 allelic variants, with a mean of 6 alleles per locus. The PIC ranged from 0.308 to 0.915. Zhao [26] conducted SSR analysis on 100 fresh maize germplasms, classifying them into six major genetic groups.

This study conducted a cluster analysis on eight major agronomic traits across 200 fresh maize inbred lines. The selected traits, consistent with the research of Tan et al. [27], exhibited highly significant variation. Following the methodology established by Marwa et al. [28], phenotypic traits served as critical criteria for germplasm evaluation. The test materials exhibited substantial genetic diversity, with clustering results categorizing them into four major groups. Notably, four benchmark inbred lines were distributed between Cluster I and Cluster III, indicating the limitations of relying solely on phenotypic clustering.

The application of SSR molecular marker technology has become increasingly prevalent in the breeding of fresh maize. SSR molecular markers are characterized by their high accuracy, enabling precise delineation of inbred lines of fresh maize. Qiu et al. [29] utilized molecular and morphological markers to examine the differences in quality and agronomic traits of fresh maize across various genetic backgrounds. Their study analyzed 41 test materials, revealing significant variation in both agronomic and quality traits, along with a high level of genetic diversity in SSR markers. The coefficients of variation for 12 agronomic and quality traits ranged from 1.72% to 36.10%, with a mean value of 14.06%. A total of 321 alleles were detected using 40 SSR markers, exhibiting polymorphic information content that varied from 0.179 to 0.866, with a mean value of 0.658. Additionally, gene diversity ranged from 0.186 to 0.877, with a mean value of 0.687. In this study, we utilized 40 pairs of SSR primers exhibiting high polymorphism and stable banding patterns to analyze the genetic diversity of 200 fresh maize inbred lines. A total of 230 allelic loci were detected, with the number of alleles per primer pair ranging from 2 to 9, averaging 5.75. The mean value of the polymorphism information content (PIC) was found to be 0.70. Among the primers, umc2105g1 and bnlg2305g1 exhibited the highest number of allelic loci. Notably, bnlg2305g1 not only had the highest number of allelic sites but also recorded the highest Polymorphic Information Content (PIC) value. This suggests that SSR molecular marker technology is particularly effective in detecting genetic polymorphisms in maize [30]. In this study, 200 fresh maize inbred lines with diverse genetic backgrounds were classified into four primary clusters through UPGMA cluster analysis. Comparison with phenotypic clustering revealed that SSR molecular marker technology is more accurate in classifying maize inbred line taxa. Additionally, the classification of self-inherited line germplasm using this marker can categorize maize germplasm resources from various sources into distinct lineages, thereby providing a foundation for future hybrid breeding. Numerous studies have demonstrated that SSR marker technology is extensively utilized in the fields of plant genetics and germplasm resource research, providing a valuable reference for subsequent related investigations [31,32,33].

The estimation of genetic distance is a crucial aspect of the comprehensive study of crop type origins and the elucidation of kinship relationships among populations [34,35]. The genetic distance analysis of 200 fresh maize inbred lines revealed that the inbred lines GM013 and GM021 share a closer kinship relationship and genetic background. In contrast, the inbred lines GM176 and GM184 exhibited significant genetic divergence, indicating that they are more distantly related and may serve as effective parental materials for the breeding of new maize types. There are significant genetic differences between the autografts GM176 and GM184, indicating their more distant relationship, enhanced hybrid vigor, and greater genetic diversity. These characteristics suggest that they can serve as parental lines for the cultivation of new maize autograft types [36,37].

## 5. Conclusions

A clustering analysis was conducted on eight phenotypic traits of 200 fresh maize inbred lines, resulting in the classification of the materials into four major clusters. The first cluster comprised 25 inbred lines, including the benchmark lines GM002 (Ye 478) and GM003 (Dan 340). The second cluster contained 38 inbred lines, while the third cluster encompassed 97 inbred lines, including the benchmark lines GM007 M017 and GM171 Qi 319. The fourth cluster included 40 inbred lines. Notably, the third cluster represented 48.5% of the total inbred lines analyzed.

A total of 230 allelic loci were detected using SSR molecular markers, with an average of 5.75 alleles identified per primer pair. The mean polymorphism information content (PIC) value was found to be 0.70. Genetic distance analysis revealed significant differences among the self-inbred lines, with GM013 and GM021 exhibiting the smallest genetic distance, while GM176 displayed the largest genetic distance from GM184.

SSR clustering divided the materials into four major clusters, with the benchmark inbred lines GM002, GM003, GM007, and GM171 distributed across different clusters. Compared to phenotypic clustering, the SSR clustering results demonstrated a higher reliability in effectively differentiating distinct clusters. Additionally, 200 fresh maize inbred lines exhibited significant genetic diversity.

## Figures and Tables

**Table 1:** Statistical parameters of different agronomic traits of 200 fresh maize inbred lines.

Traits	Minimum Value	Maximum Value	Mean Value
Plant height (cm)	104	236	172
Ear height (cm)	28	114	67
Tassel length (cm)	8	46	28
Tassel branch number	3	22	11
Ear length (cm)	8.1	22.4	13.3
Ear diameter (cm)	2.6	6.4	4
Kernel row number	8	20	14
Kernel number per row	6	36	21

**Table 2:** Results of phenotypic clustering analysis.

Forms	Quantities	Serial Number
Cluster I	25	GM003, GM127, GM121, GM166, GM145, GM065, GM087, GM156, GM151, GM119
		GM012, GM022, GM048, GM002, GM100
		GM062, GM035, GM105, GM118, GM103, GM014, GM168, GM110, GM097, GM131
Cluster II	38	GM005, GM191, GM104, GM178, GM132, GM199, GM194, GM040, GM137, GM130, GM150, GM135, GM038, GM039, GM163
		GM021, GM197, GM123, GM006, GM124, GM095, GM115, GM013, GM052, GM183, GM044, GM154, GM180, GM093, GM175, GM167, GM117, GM096, GM147, GM126, GM133, GM112, GM024
Cluster III	97	GM004, GM187, GM184, GM016, GM158, GM140, GM091, GM153, GM051, GM182
		GM200, GM160, GM193, GM060, GM086
		GM050, GM078, GM069, GM059, GM165, GM098, GM071, GM129, GM076, GM089
		GM125, GM043, GM136, GM056, GM088,
		GM007, GM042, GM084, GM063, GM161, GM192, GM141, GM142, GM128, GM054, GM047, GM067, GM173, GM020, GM198
		GM177, GM181, GM170, GM106, GM079,
		GM169, GM116, GM157, GM143, GM066, GM171, GM046, GM027, GM138, GM196
		GM113, GM190, GM111, GM108, GM070
		GM149, GM102, GM072, GM008, GM068
		GM073, GM034, GM188, GM011, GM001, GM122, GM009, GM036, GM061, GM152, GM075, GM146, GM172, GM179, GM164
		GM189, GM120, GM015, GM159, GM134, GM185, GM109, GM053, GM041, GM037, GM058, GM083
Cluster IV	40	GM025, GM186, GM176, GM174, GM162, GM139, GM045, GM017, GM057, GM107, GM031, GM101, GM155, GM090, GM092
		GM055, GM064, GM195, GM081, GM029, GM019, GM099, GM080, GM023, GM148, GM094, GM030, GM049, GM032, GM082
		GM077, GM144, GM085, GM026, GM018
		GM028, GM114, GM074, GM033, GM010

**Table 3:** Pairwise genetic distance matrix among fresh maize inbred Lines (GM010–GM023).

Number	GM-019	GM-020	GM-021	GM-022	GM-023
GM-010	0.454				
GM-011	0.561	0.471			
GM-012	0.522	0.502	0.411		
GM-013	0.509	0.571	0.308	0.702	
GM-014	0.421	0.405	0.476	0.559	0.629

Note: The smallest genetic distance was observed between GM013 and GM021.

**Table 4:** Pairwise genetic distance matrix among fresh maize inbred Lines (GM174–GM186).

Number	GM-182	GM-183	GM-184	GM-185	GM-186
GM-174	0.475	0.661	0.713	0.622	0.578
GM-175	0.562	0.572	0.477	0.532	0.607
GM-176	0.595	0.829	0.902	0.658	0.798
GM-177	0.696	0.611	0.489	0.797	0.574
GM-178	0.498	0.597	0.632	0.512	0.482

Note: The largest genetic distance was also observed between GM176 and GM184.

**Table 5:** Allelic and polymorphic information content of fresh maize detected by SSR.

Number	Primer Name	Chromosomal Location	Number of Polymorphic Bands	Effective Number of Alleles	Gene Diversity	Polymorphism Information Content
1	bnlg439g1	1.03	8	6.25	0.84	0.73
2	umc1335g2	1.06	4	2.85	0.63	0.31
3	umc1147g3	1.07	7	5.01	0.82	0.68
4	bnlg1671g4	1.1	6	3.99	0.74	0.61
5	phi96100g3	2.01	7	4.89	0.79	0.72
6	umc2007g2	2.04	5	4.03	0.62	0.58
7	umc1536g4	2.07	3	3.19	0.75	0.69
8	bnlg1940g1	2.08	8	6.99	0.88	0.79
9	bnlg1520g4	2.09	4	4.72	0.81	0.73
10	umc2105g1	3	9	7.02	0.86	0.82
11	phi053g2	3.05	7	5.18	0.77	0.7
12	umc1489g3	3.07	6	3.98	0.78	0.76
13	phi072g1	4	4	3.76	0.71	0.64
14	bnlg490g3	4.04	5	3.99	0.81	0.74
15	bnlg2291g2	4.07	4	3.04	0.67	0.56
16	umc1999g3	4.09	7	5.94	0.77	0.74
17	umc2115g4	5.02	5	5.01	0.75	0.71
18	umc1705g2	5.03	7	6.96	0.82	0.79
19	umc1429g3	5.03	8	7.92	0.84	0.81
20	bnlg2305g1	5.07	9	6.32	0.86	0.83
21	bnlg161g2	6	5	4.99	0.78	0.75
22	bnlg249g4	6.01	7	6.38	0.84	0.8
23	bnlg1702g1	6.05	6	5.63	0.78	0.73
24	phi299852g3	6.07	5	4.97	0.76	0.72
25	umc1545g1	7	6	5.73	0.75	0.71
26	umc2160g4	7.01	5	3.95	0.75	0.71
27	umc1936g4	7.03	6	5.28	0.76	0.74
28	umc1125g2	7.04	5	4.76	0.77	0.74
29	bnlg2235g4	8.02	7	6.46	0.81	0.79
30	bnlg240g1	8.06	8	7.51	0.83	0.81
31	phi080g2	8.08	6	5.39	0.79	0.76
32	phi233376g3	8.09	5	4.65	0.74	0.72
33	umc2084g3	9.01	6	5.51	0.78	0.75
34	phi065g1	9.03	4	3.74	0.73	0.7
35	umc1492g2	9.04	3	1.75	0.42	0.3
36	umc1231g4	9.05	6	4.86	0.78	0.73
37	phi041g4	10	2	1.71	0.47	0.42
38	umc1432g1	10.02	3	2.21	0.62	0.58
39	umc2163g3	10.04	5	4.68	0.76	0.71
40	umc1506g2	10.05	7	6.01	0.82	0.79

**Table 6:** Results of SSR molecular marker clustering.

Forms	Quantities	Serial Number
Cluster I	53	GM001, GM003, GM005, GM017, GM023, GM024, GM030, GM036, GM044, GM050, GM052, GM053, GM055, GM058, GM066, GM072, GM075, GM080, GM085, GM086, GM087, GM088, GM090, GM091, GM095, GM097, GM099, GM100, GM106, GM109, GM111, GM112, GM115, GM122, GM125, GM130, GM133, GM140, GM143, GM147, GM148, GM152, GM153, GM161, GM165, GM168, GM175, GM177, GM178, GM181, GM188, GM190, GM192
Cluster II	102	GM002, GM004, GM006, GM008, GM009, GM011, GM013, GM014, GM015, GM016, GM018, GM019, GM020, GM021, GM022, GM026, GM027, GM028, GM029, GM031, GM033, GM034, GM035, GM038, GM039, GM041, GM042, GM046, GM047, GM049, GM054, GM056, GM057, GM060, GM061, GM062, GM063, GM065, GM067, GM069, GM071, GM074, GM077, GM078, GM079, GM082, GM083, GM084, GM094, GM096, GM098, GM102, GM103, GM104, GM110, GM113, GM117, GM118, GM119, GM120, GM121, GM123, GM124, GM126, GM127, GM128, GM131, GM134, GM135, GM136, GM137, GM138, GM144, GM145, GM146, GM151, GM156, GM159, GM162, GM163, GM164, GM166, GM167, GM169, GM172, GM176, GM179, GM180, GM182, GM183, GM185, GM187, GM189, GM191, GM193, GM194, GM195, GM196, GM197, GM198, GM199, GM200
Cluster III	28	GM007, GM010, GM012, GM025, GM032, GM037, GM040, GM045, GM048, GM051, GM059, GM068, GM070, GM073, GM076, GM089, GM092, GM093, GM101, GM105, GM107, GM108, GM114, GM116, GM132, GM149, GM150, GM160
Cluster IV	17	GM043, GM064, GM081, GM129, GM139, GM141, GM142, GM154, GM155, GM157, GM158, GM170, GM171, GM173, GM174, GM184, GM186

## Data Availability

The original contributions presented in this study are included in the article. Further inquiries can be directed to the corresponding author.

## Abbreviations

The following abbreviations are used in this manuscript:

SSR	Simple Sequence Repeat
PIC	Polymorphism Information Content
GS	Genetic Similarity Coefficient
GD	Genetic Distance

## Appendix A

**Table A1:** Mean traits of 200 fresh maize inbred lines.

Number	Plant Height (cm)	Ear Height (cm)	Tassel Length (cm)	Tassel Branch Number (Number)	Ear Length (cm)	Ear Diameter (cm)	Kernel Row Number (Rows)	Kernel Number Per Row (Kernels)
GM001	184	69	28	6	8.2	3.9	16	21
GM002	176	61	21	7	14	4	14	20
GM003	165	54	26	5	6	5.3	18	18
GM004	195	72	31	12	15.3	4.9	12	18
GM005	155	52	19	11	12.2	3.9	14	16
GM006	111	35	11	4	12.9	3.3	12	14
GM007	190	58	27	9	16	4.8	16	23
GM008	145	55	35	12	9.6	4.3	12	16
GM009	168	63	21	18	15.2	3.1	14	15
GM010	175	68	31	17	16.1	4.2	18	22
GM011	180	71	31	16	18.1	3.6	18	28
GM012	146	58	28	9	13.2	4.1	14	18
GM013	134	40	24	4	11.6	3.7	12	14
GM014	168	57	26	10	11.1	4.3	14	20
GM015	197	65	31	12	14.1	4.2	14	18
GM016	210	85	29	4	11.9	4.6	18	20
GM017	221	95	30	17	16.8	4.8	16	25
GM018	187	65	30	15	17	4.6	14	25
GM019	186	89	29	16	18.2	3.1	14	22
GM020	145	50	27	12	13.4	4.2	12	24
GM021	123	52	17	5	7.8	3.2	12	15
GM022	156	54	28	8	12.5	4	14	16
GM023	176	77	30	16	20	3.5	16	32
GM024	126	40	25	6	12.8	2.6	14	18
GM025	190	83	35	12	17.5	3.5	12	28
GM026	195	70	32	14	17.1	4.3	14	26
GM027	167	61	31	8	12.5	3.1	12	19
GM028	184	70	25	14	18.2	4.5	16	28
GM029	182	91	32	14	18.7	3.1	16	27
GM030	196	79	33	10	16.2	3.8	14	24
GM031	221	88	31	20	16.5	4.6	18	26
GM032	190	81	32	13	16.4	3.5	12	26
GM033	185	70	35	15	17.7	3.8	16	23
GM034	175	75	33	9	13.3	3.9	16	22
GM035	164	60	30	7	10.3	4.2	12	19
GM036	155	51	33	9	17.2	3.9	16	24
GM037	143	47	30	11	15.7	3.7	18	22
GM038	134	55	18	11	14	3.2	14	20
GM039	170	56	11	9	10	3	12	16
GM040	172	60	20	13	10	3.4	14	18
GM041	169	66	35	9	12.2	3.6	16	25
GM042	210	65	27	15	14.5	4.6	12	16
GM043	205	72	32	15	13.5	4	14	15
GM044	118	35	21	7	11.5	3.9	14	16
GM045	215	85	32	16	17.3	4.8	16	25
GM046	164	65	31	6	10.6	3.4	12	18
GM047	174	75	29	9	12.2	4.1	14	26
GM048	176	71	21	10	13	4.2	14	20
GM049	198	85	35	13	15.5	4.1	14	24
GM050	183	101	28	11	13.4	4	16	23
GM051	224	86	37	13	18.1	4.4	16	28
GM052	124	46	25	8	11.8	3.7	12	20
GM053	168	64	32	7	11.5	3.2	14	19
GM054	178	75	31	9	12.1	4	14	22
GM055	198	94	37	21	18.5	4.1	16	32
GM056	201	71	28	10	14.5	3.6	14	16
GM057	210	87	29	16	15.1	5.1	18	25
GM058	185	67	24	9	12.9	4.9	16	28
GM059	216	89	25	14	13.9	4.2	12	30
GM060	226	83	37	8	16	4.4	14	23
GM061	138	36	21	9	16.2	4.1	14	24
GM062	158	75	31	6	9.8	4.6	12	18
GM063	195	95	36	8	11	3.8	14	16
GM064	210	109	40	23	16.3	4.5	18	35
GM065	172	80	29	10	11.8	4.4	16	22
GM066	143	52	26	9	11.3	3.1	10	28
GM067	161	63	29	8	12.2	4.1	14	24
GM068	171	69	33	9	11	3.9	16	24
GM069	186	102	26	10	12.8	4.1	14	27
GM070	156	56	35	12	13.5	3.6	14	16
GM071	194	66	34	14	15.6	4.3	14	20
GM072	153	54	33	15	12.7	3.1	12	16
GM073	173	81	32	9	13	4	16	22
GM074	189	72	31	14	16.8	3.9	16	27
GM075	170	65	22	7	14.2	4.7	14	26
GM076	191	67	33	12	13.5	4.2	12	18
GM077	195	95	35	13	18.9	4.1	12	24
GM078	187	102	31	13	12.4	3.9	14	23
GM079	144	51	29	11	14.1	3.5	12	26
GM080	194	87	33	17	15.3	3.5	14	28
GM081	204	100	33	19	13	4.1	18	33
GM082	206	85	37	17	17.8	3.8	12	24
GM083	187	62	26	11	17.3	5.4	16	30
GM084	193	98	34	7	13	3.5	12	18
GM085	185	73	38	16	18.2	3.9	14	28
GM086	181	98	31	11	13.2	4.3	16	25
GM087	154	73	30	8	10	4.1	16	19
GM088	197	70	30	13	14.5	4.7	14	18
GM089	214	83	35	13	13.6	3.9	12	18
GM090	228	103	35	20	15.3	4.1	16	30
GM091	189	96	29	14	12.3	4.7	18	27
GM092	211	99	37	23	16.7	4	16	33
GM093	119	42	27	11	13.4	3.6	14	18
GM094	176	76	31	12	13.7	3.6	14	24
GM095	128	40	25	7	10.4	3.8	12	16
GM096	114	42	23	11	12.1	3.5	12	16
GM097	119	58	26	9	13.7	4.9	16	20
GM098	223	69	40	13	15.5	3.9	14	14
GM099	200	81	35	16	16.2	3.3	16	24
GM100	149	56	22	7	13.6	3.9	14	22
GM101	189	81	30	23	19.2	4.5	16	28
GM102	178	61	37	12	11.3	3.7	12	14
GM103	170	63	25	9	11	4.7	14	20
GM104	132	65	22	11	9.5	3.6	14	18
GM105	155	79	26	6	9.5	4.7	14	15
GM106	147	56	25	11	14.2	3.6	12	24
GM107	229	99	29	19	17	4.5	18	32
GM108	165	63	31	9	12.6	3.6	14	17
GM109	168	61	34	7	11.1	3.3	12	18
GM110	157	60	20	3	10.9	4.4	16	15
GM111	160	60	33	6	12.3	3.2	14	17
GM112	118	41	24	9	10.1	3.3	10	14
GM113	181	73	27	6	12.9	3.2	12	22
GM114	190	83	25	17	16.9	3.9	14	24
GM115	135	40	25	5	11.3	3.6	12	16
GM116	146	50	24	10	11.2	3.7	12	26
GM117	118	39	24	10	13.4	3.9	12	16
GM118	166	54	26	9	10	4.7	14	21
GM119	152	61	26	9	12.3	4.3	14	19
GM120	180	60	38	13	14.6	4.4	14	17
GM121	159	64	25	9	13.3	4.3	16	20
GM122	206	63	23	15	14.3	3.5	14	14
GM123	124	56	15	5	9.1	3.8	14	17
GM124	127	44	26	7	12.1	3.9	12	15
GM125	196	71	29	15	13.5	4.2	14	18
GM126	149	43	27	6	10.2	3.8	10	15
GM127	156	60	26	9	13.5	4.5	16	20
GM128	179	78	32	9	12	4.1	14	24
GM129	182	65	36	12	15.3	4.2	12	18
GM130	145	48	16	7	11.3	3.5	14	22
GM131	162	53	27	9	12	5.2	16	8
GM132	138	60	20	13	11	3.5	12	16
GM133	143	44	27	7	10.6	3.2	12	14
GM134	178	73	31	9	13.3	4.2	12	26
GM135	138	45	22	9	13.4	3.3	14	22
GM136	199	67	29	12	14	4	14	16
GM137	143	55	19	9	11	3.2	14	20
GM138	166	65	29	5	12.6	3.4	12	19
GM139	211	90	31	24	16.5	4.2	12	30
GM140	188	100	27	10	12.7	4.5	18	23
GM141	155	69	22	11	12.6	3.8	12	26
GM142	177	70	24	10	11	4.1	12	31
GM143	148	55	26	9	13.6	3.4	10	27
GM144	195	79	36	13	18.5	3.9	14	31
GM145	175	69	29	10	12.2	4.4	16	19
GM146	173	62	18	9	11.5	4.7	14	24
GM147	145	45	22	5	11.2	3.4	10	15
GM148	190	79	31	14	15.7	3.9	14	26
GM149	167	63	34	9	12	3.7	12	19
GM150	126	46	16	9	12.2	3.5	14	16
GM151	169	59	28	7	11.8	4.4	16	17
GM152	171	68	22	12	14.6	4.6	14	26
GM153	223	85	39	11	16.6	4.1	16	28
GM154	121	40	26	8	11.3	3.9	14	15
GM155	211	100	30	19	17.1	3.9	16	30
GM156	160	58	29	6	13.5	4.2	16	19
GM157	135	50	25	10	13.2	3.3	10	27
GM158	213	95	32	8	12.9	4.9	14	20
GM159	170	60	22	10	11.9	4.3	12	22
GM160	198	83	30	11	16	4.3	18	28
GM161	175	64	20	11	12.4	4.1	12	22
GM162	193	92	39	17	17.6	5.2	14	32
GM163	125	51	17	7	7.5	3.1	12	15
GM164	205	79	25	12	12.9	4.7	18	20
GM165	188	67	40	11	13.6	3.9	14	16
GM166	177	70	26	11	13.8	4.3	16	22
GM167	116	37	22	12	13.3	3.7	12	16
GM168	166	56	24	5	11.3	4.9	14	18
GM169	155	56	26	10	12.5	3.3	12	25
GM170	148	59	23	8	15.1	3.6	12	24
GM171	158	54	29	11	10.5	3.4	14	26
GM172	174	69	22	13	14.3	4.2	16	26
GM173	146	57	28	13	13.9	4.1	14	24
GM174	220	87	36	15	16.9	4.7	12	34
GM175	113	39	23	13	11.2	3.4	14	16
GM176	234	90	39	15	17.2	4.6	14	30
GM177	142	56	31	12	11.4	3.9	12	22
GM178	133	61	20	10	9.6	3.8	14	16
GM179	188	69	23	9	12.9	4.9	18	24
GM180	117	33	26	12	13.5	3.7	14	15
GM181	165	59	27	11	15.2	4.2	12	19
GM182	200	84	34	13	17.1	4.2	16	26
GM183	129	43	22	7	13.2	4	14	16
GM184	206	92	31	5	12.2	4.7	16	20
GM185	145	52	29	11	14	3.5	12	29
GM186	222	88	39	15	18.2	4.5	14	32
GM187	205	93	35	5	12.3	4.3	16	20
GM188	168	65	35	9	11.1	3.1	16	19
GM189	203	79	38	12	11.7	4.6	16	28
GM190	161	60	32	5	12.4	3.4	14	19
GM191	137	65	23	13	9.4	3.5	14	16
GM192	176	67	23	12	14.3	4.3	12	24
GM193	198	91	35	7	15.3	3.9	16	22
GM194	151	60	23	9	11	3.8	14	16
GM195	213	104	37	39	13.6	4.7	18	28
GM196	169	67	28	6	10.2	3.1	12	18
GM197	125	39	14	5	9.6	3.5	12	16
GM198	152	58	30	9	12.9	4.1	12	22
GM199	147	62	21	9	11	4.3	14	16
GM200	193	85	31	8	14.5	4.2	18	26
